# Supplementary material for: Highly reversible transition metal migration in superstructure-free Li-rich oxide boosting voltage stability and redox symmetry
Source: Nat Commun. 2024 Jun 4;15:4742. doi: 10.1038/s41467-024-48890-1 (PMC11150454; doi:10.1038/s41467-024-48890-1)
Supplement: Supplementary file 1 — Supplementary Information [file 41467_2024_48890_MOESM1_ESM.pdf]

Supplementary Information

Highly reversible transition metal migration in superstructure-free Li-rich oxide boosting voltage stability and redox symmetry

Tianwei Cui,[1,6] Jialiang Xu,[2,6] Xin Wang,[1] Longxiang Liu,[3] Yuxuan Xiang,[4,5] Hong Zhu,[2] Xiang Li,[1,*] and Yongzhu Fu[1,*]

[1]College of Chemistry, Zhengzhou University, Zhengzhou 450001, China

[2]University of Michigan-Shanghai Jiao Tong University Joint Institute, Shanghai Jiao Tong University, Shanghai, 200240, China

[3]Department of Materials, University of Oxford, Parks Road, Oxford OX1 3PH

[4]Research Center for Industries of the Future, Westlake University, Hangzhou, Zhejiang 310030, China

[5]School of Engineering, Westlake University, Hangzhou, Zhejiang 310030, China.

[6]These authors contributed equally

[*]Corresponding author: xli@zzu.edu.cn

[*]Corresponding author: yfu@zzu.edu.cn

Supplementary Figures

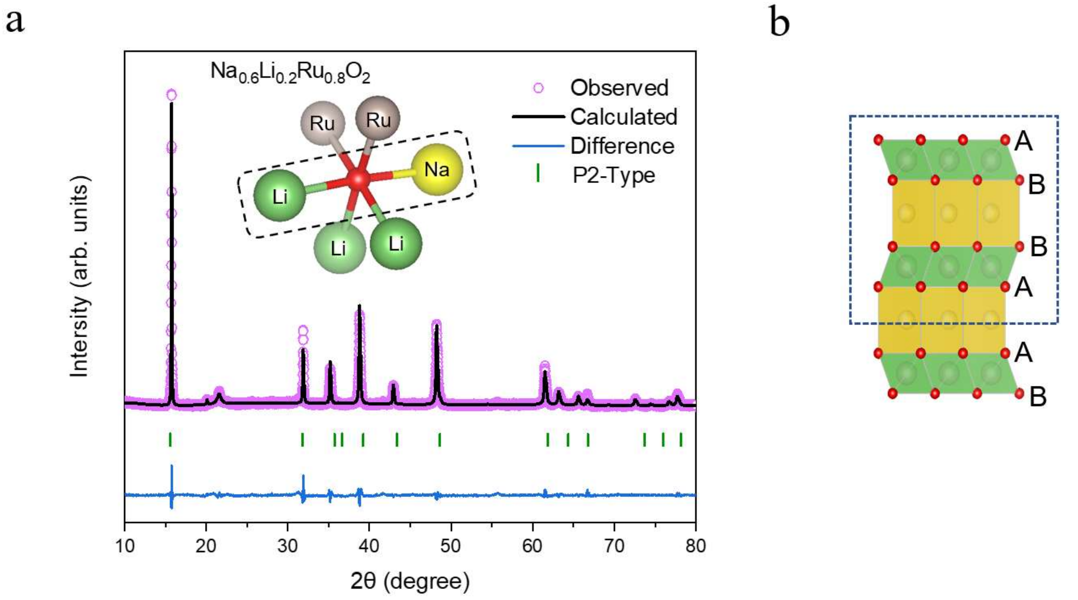

Supplementary Fig. 1. Structure of precursor P2-$Na_{0.6}Li_{0.2}Ru_{0.8}O_2$. a XRD pattern of P2-$Na_{0.6}Li_{0.2}Ru_{0.8}O_2$ with Rietveld refinement. (inset: Na-O-Li configuration). **b** Schematic P2-type “ABBA” crystal structure. Source data are provided as a Source Data file.

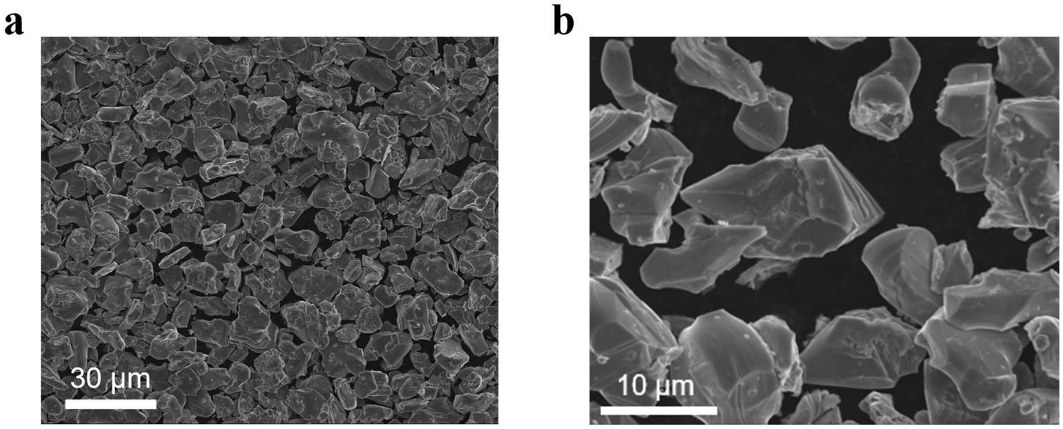

Supplementary Fig. 2. SEM image of LLRO at (**a**) large scale and (**b**) small scale.

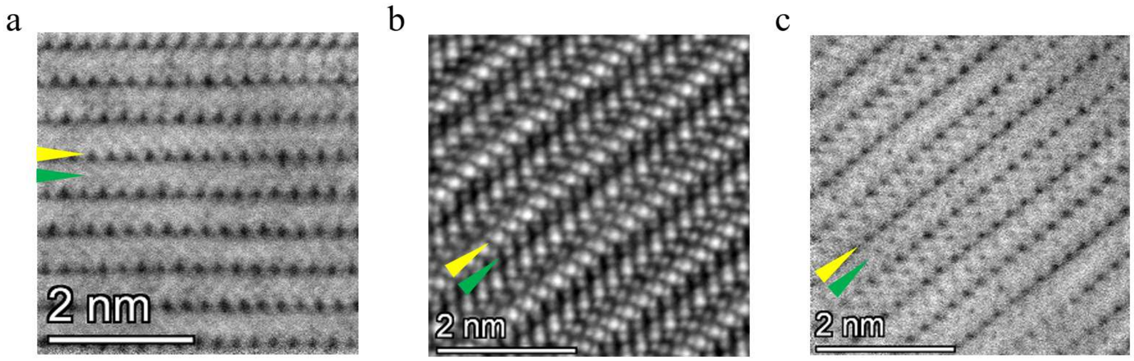

Supplementary Fig. 3. Atomic arrangement of LLRO. ABF-STEM images of (**a**) the de-lithiation state of LLRO. **b** IDPC-STEM and (**c**) ABF-STEM of discharged state of LLRO. ABF-STEM can observe light atoms such as Li and O. It is apparent that Li atoms exist at the AM layer at the pristine, and disappear at the end of the charge and reappear at the discharged state. Yellow arrow: TM layer. Green arrow: AM layer.

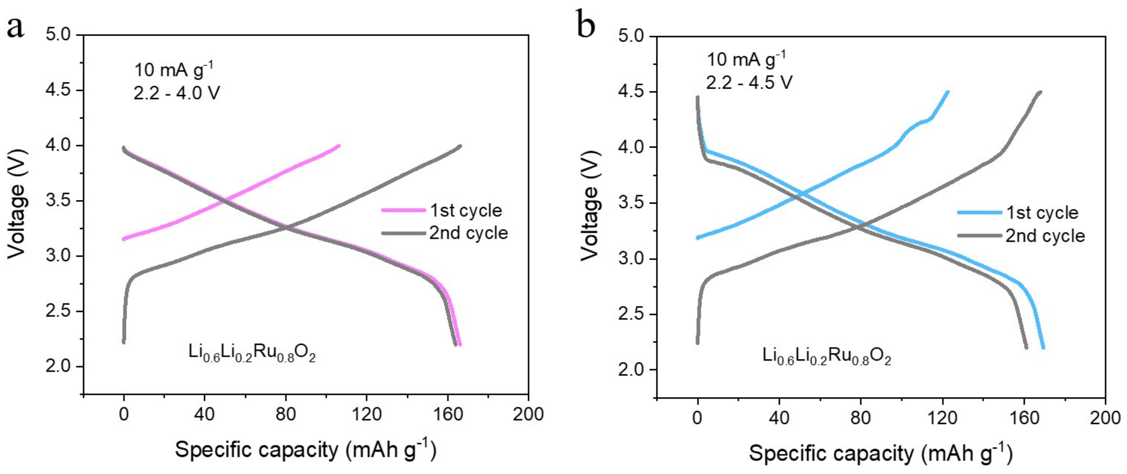

Supplementary Fig. 4. The initial two charge/discharge curves of LLRO between (**a**) 2.2–4.0 V and (**b**) 2.2–4.5 V. Source data are provided as a Source Data file.

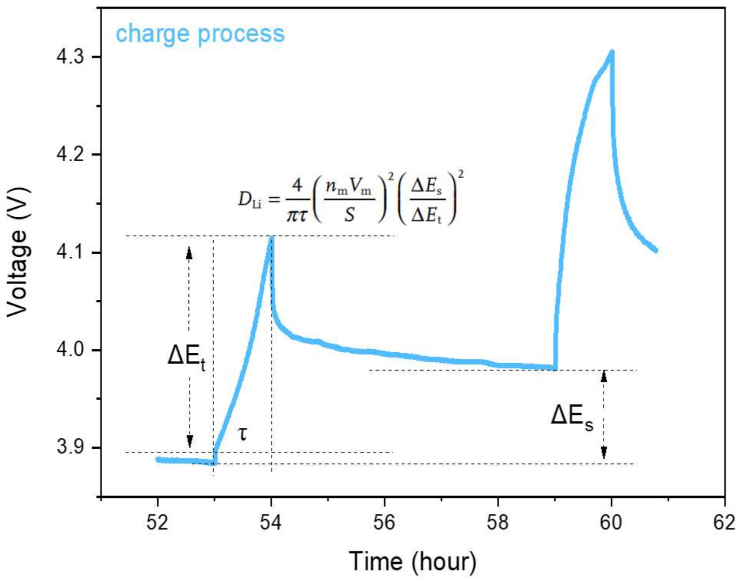

Supplementary Fig. 5. GITT parameters within the selected area, where τ is the limited time period, n_m is the mole number of the electrode, V_m is the molar volume of the LLRO, S is the area of the electrode, ΔE_s and ΔE_t are the change in the steady state potential and the total change during the current flux by deducting the IR drop, respectively.

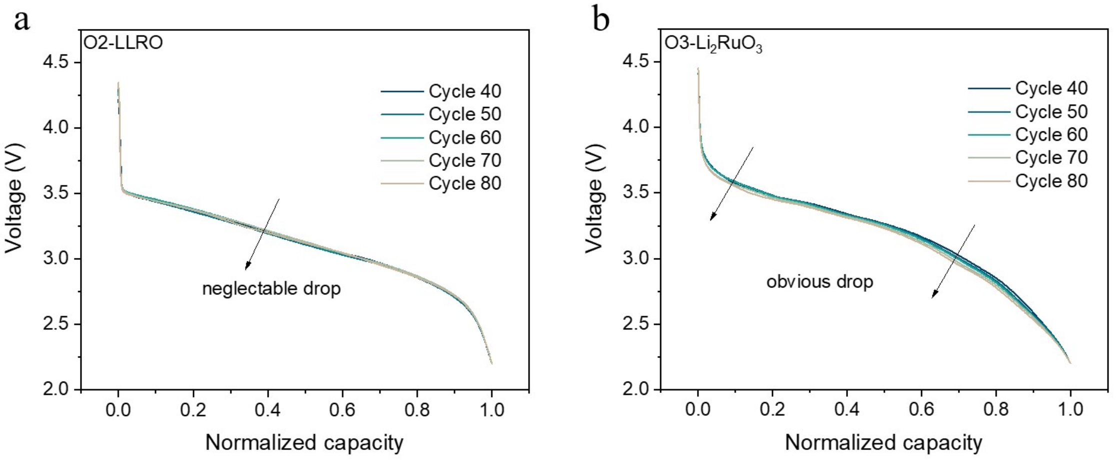

Supplementary Fig. 6. Comparison of voltage decay in two samples. The normalized capacity of (**a**) O2-LLRO and (**b**) O3-Li_2RuO_3 during cycling at 100 mA g^{-1}. Source data are provided as a Source Data file.

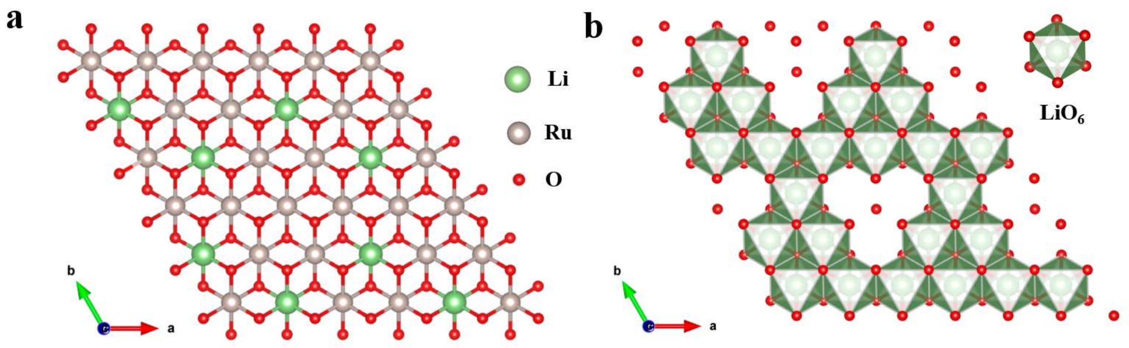

Supplementary Fig. 7. The optimized structure of LLRO. a TM layer and (**b**) Li layer, with Li and Ru randomly distributed in the TM layer according to 1:4, and vacancies in the Li layer randomly distributed with Li according to 1:2. Where we performed 3×3×1 cell expansion of the structure to get all the possible structures under the $Li_{16}Ru_{14}O_{36}$ composition, arranged according to Ewald energy from smallest to largest and calculated the DFT structure energy to get the most stable structure.

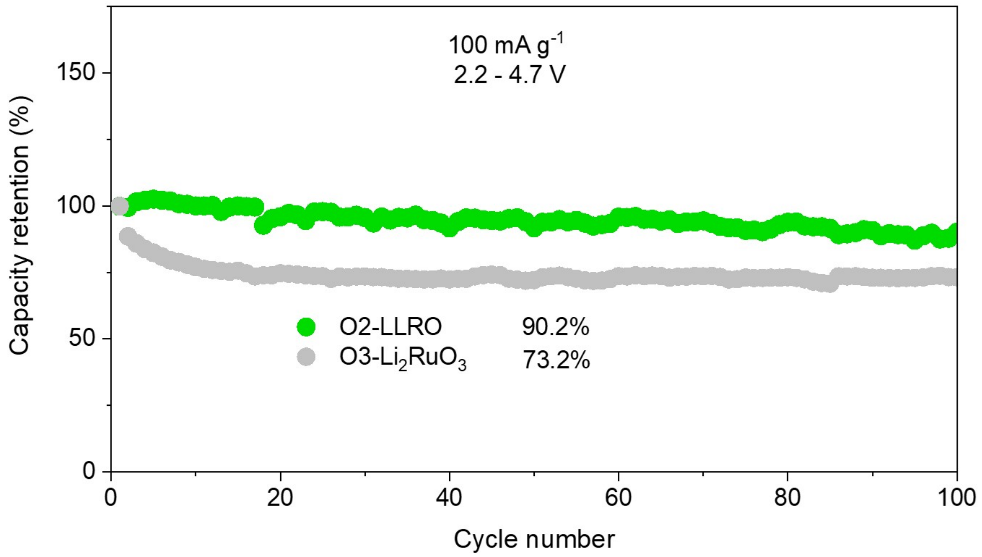

Supplementary Fig. 8. Comparison of the capacity retention of O2-LLRO and O3-Li_2RuO_3. Source data are provided as a Source Data file.

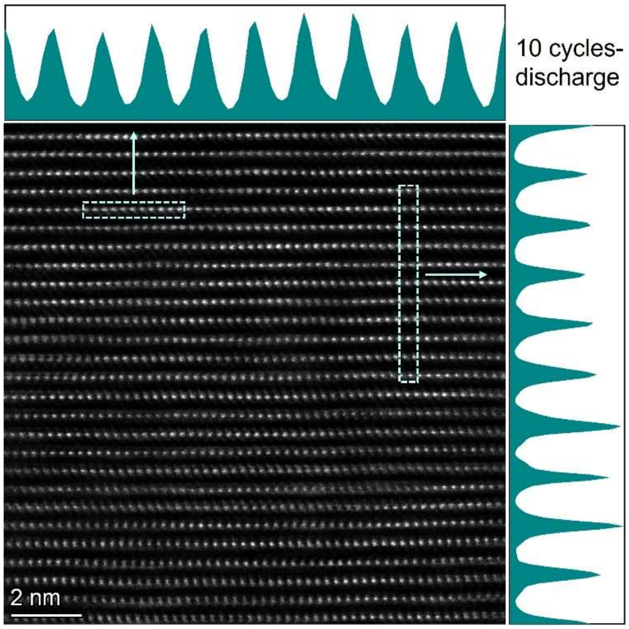

Supplementary Fig. 9. HAADF-STEM of O2-LLRO after 10 cycles.

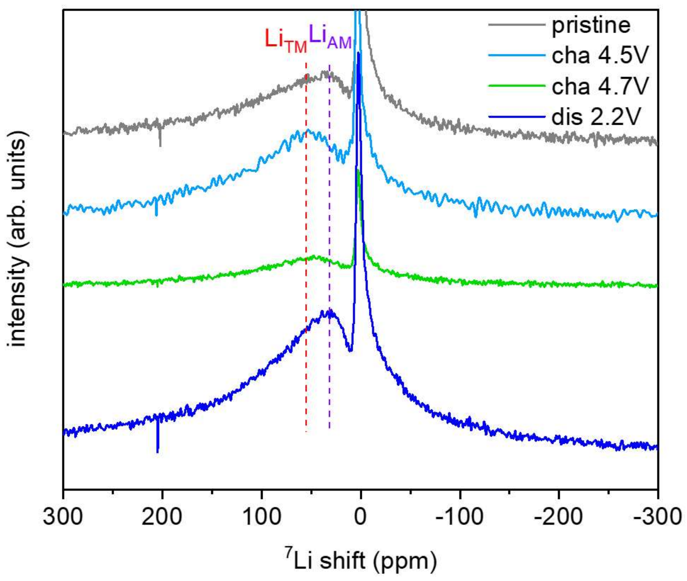

Supplementary Fig. 10. ^{7}Li solid-state NMR at different states. Source data are provided as a Source Data file.

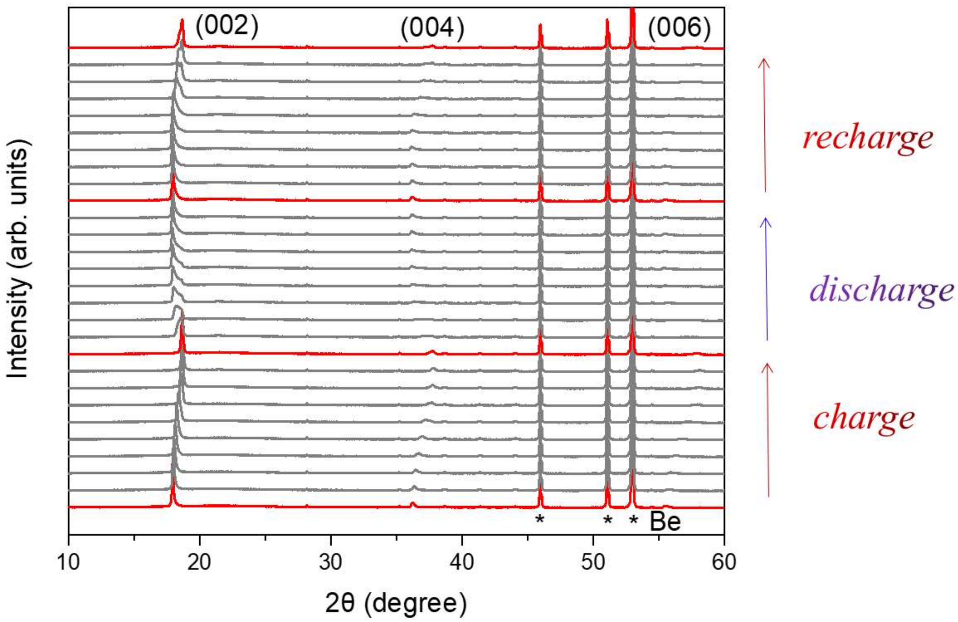

Supplementary Fig. 11. In-situ XRD of LLRO during the initial cycles. Be foil is marked as an asterisk. Source data are provided as a Source Data file.

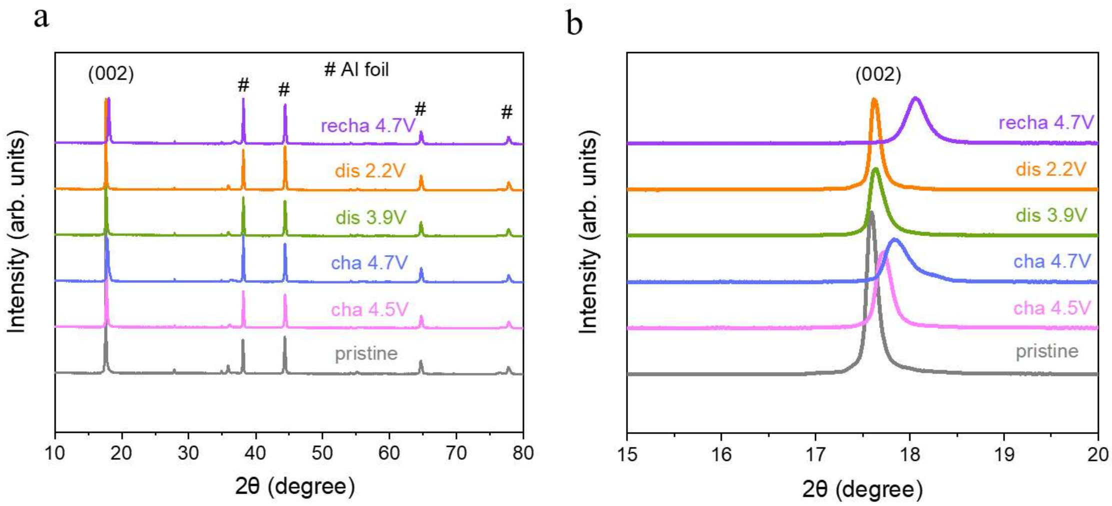

Supplementary Fig. 12. Ex-situ XRD of LLRO. a Ex-situ XRD of LLRO at different states. **b** Enlarged area of (002) peak. Source data are provided as a Source Data file.

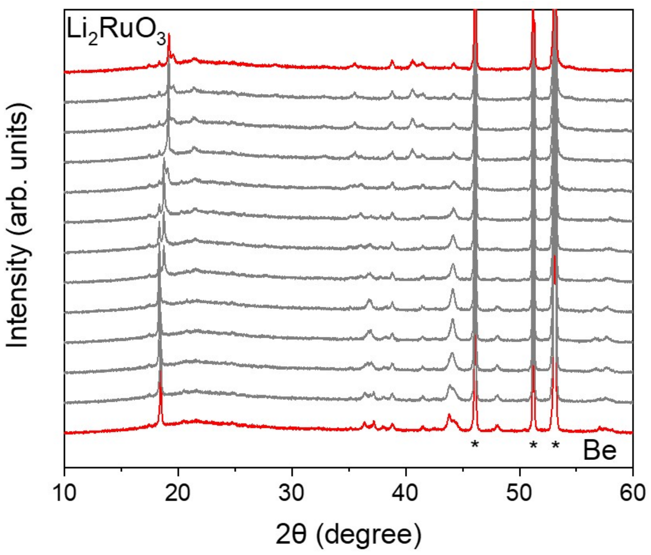

Supplementary Fig. 13. In-situ XRD patterns of O3-Li_2RuO_3 for the initial charge process. Source data are provided as a Source Data file.

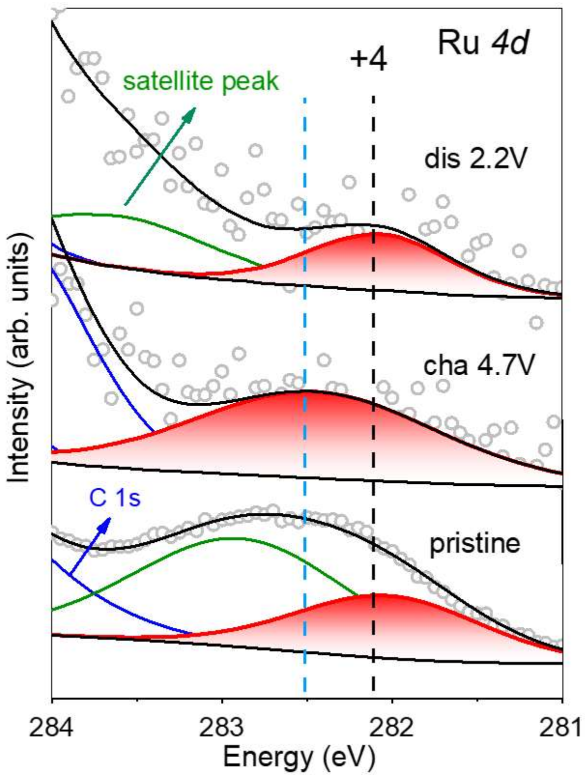

Supplementary Fig. 14. Ru *4d* XPS spectra of LLRO at the pristine, charged, and discharged state. The black and blue dotted lines indicate +4 and +5 valence of Ru. Source data are provided as a Source Data file.

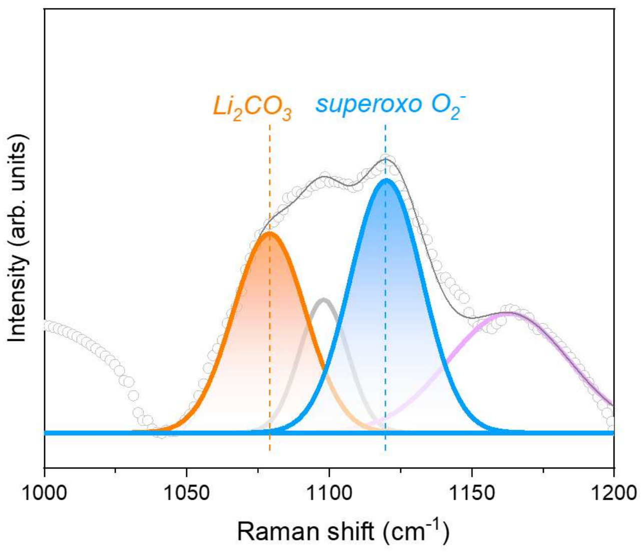

Supplementary Fig. 15. Raman spectra at the end of charge at around 1100 cm^{-1}. The orange line represents Li_2CO_3 residual on the surface of the cathode. The blue line represents superoxo-species. Source data are provided as a Source Data file.

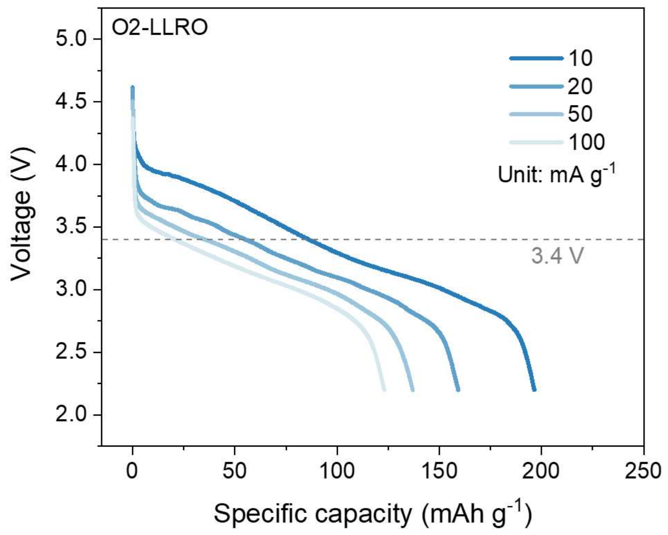

Supplementary Fig. 16. Discharge curves of O2-LLRO at different current densities. Source data are provided as a Source Data file.

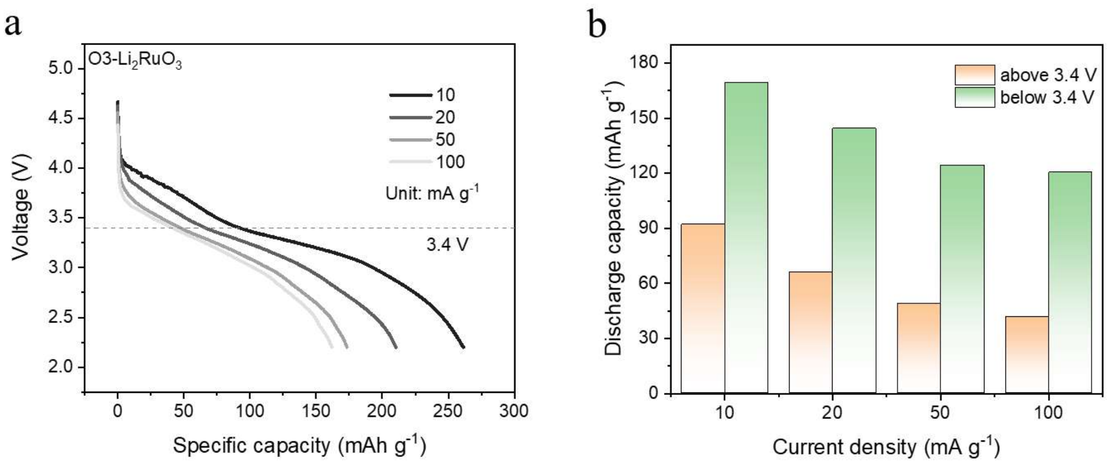

Supplementary Fig. 17. Oxygen redox asysmmetry of Li_2RuO_3. a Discharge curves of O3-Li_2RuO_3 at different current densities. **b** Discharge capacity for the two regions (2.2–3.4 V and 3.4–4.7 V) upon the increased current densities. Source data are provided as a Source Data file.

Supplementary Tables

Supplementary Table 1. Refined crystallographic parameters by Rietveld analysis for $Na_{0.6}Li_{0.2}Ru_{0.8}O_2$. 194 space group *$P6_3/mmc$*, a = b = 2.94626 Å, c = 11.21904 Å, V = 84.13 Å^3, α = β = 90°, γ = 120°.

Atom	x	y	z	Occupancy
Na1	0.00000	0.00000	0.25000	0.3
Na2	0.33333	0.66667	0.25000	0.3
Li	0.00000	0.00000	0.00000	0.2
Ru	0.00000	0.00000	0.00000	0.8
O	0.33333	0.66667	0.08868	1.0

Supplementary Table 2. Refined crystallographic parameters by Rietveld analysis for $Li_{0.6}Li_{0.2}Ru_{0.8}O_2$. 186 space group $P6_3/mc$, a = b = 2.83750 Å, c = 9.65640 Å, V = 69.33 Å^3, α = β = 90°, γ = 120°.

**Atom**	**x**	**y**	**z**	**Occupancy**
Li1	0.66667	0.33333	0.00000	0.2
Li2	0.33333	0.66667	0.24126	0.6
Ru	0.66667	0.33333	0.00000	0.8
O1	0.33333	0.00000	0.09300	1.0
O2	0.00000	0.00000	0.40700	1.0

Supplementary Table 3. Calculation details. Relative site energy of intermediate and final sites calculated along the four possible migration paths of Ru1 and Ru2 for structure 1.

Site	Ru1-OOT	Ru1-OTO	Ru2-OOT	Ru2-OTO
Initial (eV)	0	0	0	0
Intermedia (eV)	-0.33798	0.62684	-0.74723	-0.0064
Final (eV)	1.94584	1.14138	0.9865	-0.23195

Supplementary Table 4. Calculation details. Relative site energy of intermediate and final sites calculated along the four possible migration paths of Ru1 and Ru2 for structure 2.

Site	Ru1-OOT	Ru1-OTO	Ru2-OOT	Ru2-OTO
Initial (eV)	0	0	0	0
Intermedia (eV)	0.9276	0.75589	-0.73604	-0.19881
Final (eV)	1.8064	1.08384	0.05074	-0.03379
